# Case report: Brincidofovir-induced reversible severe acute kidney injury in 2 solid-organ transplant for treatment of cytomegalovirus infection

**DOI:** 10.1097/MD.0000000000005226

**Published:** 2016-11-04

**Authors:** Emmanuel Faure, Tatiana Galperine, Olivier Cannesson, Sophie Alain, Viviane Gnemmi, Celine Goeminne, Annie Dewilde, Johana Béné, Mohamed Lasri, Célia Lessore de Sainte Foy, Arnaud Lionet

**Affiliations:** a Service de Néphrologie et Transplantation; b Unité des maladies infectieuses, Hôpital Huriez, CHU de Lille, Lille; c Centre National de Référence du Cytomégalovirus, CHU de Limoges, Limoges; d Institut de Pathologie, Centre Biologie-Pathologie; e Hôpital Cardiologique; f Institut de microbiologie, Laboratoire de Virologie, Centre Biologie-Pathologie, CHU de Lille; g Centre Régional de Pharmacovigilance du Nord-Pas de Calais, CHU Lille; h Pharmacie hospitalière, CHRU de Lille, Lille, France.

**Keywords:** brincidofovir, CMV infection, nephrotoxicity, solid-organ transplantation

## Abstract

**Rationale:**

Resistant cytomegalovirus-mediated infections are increasing in solid organ recipient with few available alternative treatments. Brincidofovir is an oral broad-spectrum antiviral in development for prevention and treatment of viral infection, particularly cytomegalovirus.

**Patients concerns:**

Although brincidofovir is an analogue of cidofovir, previous studies reported no renal toxicity.

**Diagnoses:**

Here, we report 2 cases of severe tubular necrosis in solid organ recipients, 1 heart and 1 kidney transplant.

**Interventions:**

Both patients received brincidofovir for the treatment of cytomegalovirus infection with mutation of UL-97. They presented an acute kidney injury without any occurrence of other clinical event such as introduction of nephrotoxic drug, graft rejection, urinary tract obstruction or infection, and calcineurin inhibitor overdosage. In each case, renal biopsy showed extended tubular necrosis.

**Outcomes:**

The discontinuation of brincidofovir led to improve renal function without other any intervention. Reintroduction of brincidofovir in case 1, due to the absence of other medical alternative, led to a new episode of acute kidney injury. One more time, renal biopsy showed tubular necrosis and patient recovered renal function after discontinuation.

**Lessons:**

To our knowledge, this is the first report of brincidofovir-mediated renal adverse event. Clinicians may be aware of this severe complication in this specific population.

## 1 Introduction

Brincidofovir (CMX-001) is a recent broad-spectrum antiviral agent with an in vitro activity against polyomaviruses, poxvirus, adenoviruses, herpesviruses, and particularly cytomegalovirus (CMV).^[1]^ Brincidofovir is a lipid-conjugated analogue of known intravenous cidofovir^[2]^ allowing an increased oral bioavailability and a prolonged half-life.^[3]^ Given its pharmacologic properties, brincidofovir is not a target of human organic transporter^[4]^ and exhibits a reduced nephrotoxicity compared with cidofovir. Phase II study demonstrated that patients receiving 200 mg weekly, for CMV prophylaxis, showed a decreased incidence of CMV infection.^[4]^ A currently phase III study in stem-cell transplantation did not show any difference in renal adverse effect between patients receiving either brincidofovir 100 mg twice weekly or placebo (clinicaltrials.gov NCT01769170). Brincidofovir has failed phase III trial in hematopoietic stem cell transplantation; for this reason, studies in solid organ transplantation are currently halted. Nevertheless, in France, Brincidofovir may be obtained by the French national drug agency (*Agence national de santé du medicament*, ANSM) for compassionate use. The criteria are a documented resistant CMV-infection with no other therapeutic option (especially mutations in the *CMV UL97* kinase gene), with a coinfection by another Herpesviridae. In 2015, in our center, 2 patients were treated with brincidofovir for CMV infection with UL97 mutation after solid organ transplantation. The 2 patients exhibited a brincidofovir-induced nephrotoxicity requiring the drug discontinuation.

### 1.1 Case 1

**Figure 1:**
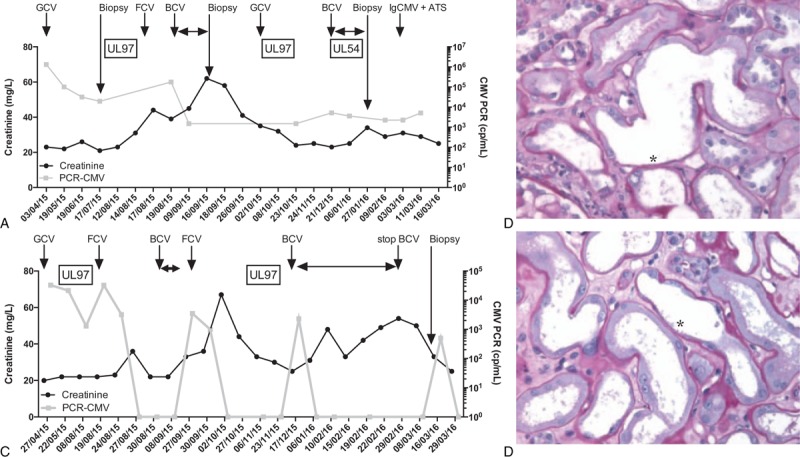
Clinical and morphological features of brincidofovir-induced nephropathy in the 2 cases. A–C, Clinical features of the first case (A) and the second case (C). ATS = artesunate, BCV = Brincidofovir, Biopsy = native or graft kidney biopsy, FCV = Foscarnet, GCV = Ganciclovir relayed with Valganciclovir, IgCMV = Immunoglobulin anti-CMV. Viral mutation assessments are reported in boxes. Grey line corresponds to cytomegalovirus (CMV) viral load assessed by quantitative polymerase chain reaction; black line corresponds to creatinin level. (B–D) Morphological features: nephrotoxic acute tubular injury observed in 2 cases, B case no.1, D case no. 2. Kidney biopsies displayed diffuse and severe acute tubular necrosis, characterized by tubular dilation and flattening of tubular epithelium with loss of brush border (not detectable here on PAS stain), cell necrosis with gap along the tubular basement membrane (∗). Notably, interstitial and tubular inflammations are not detected. PAS, Magnification, ×400.

The first patient is a 59-year-old man who received a second renal transplantation for malformative nephropathy in February 2015. The immunosuppressive therapy associated tacrolimus 4 mg per day (0.04 mg/kg per day, T0 = 5 μg/L) (calcineurin inhibitor, CNI), everolimus (1.5 mg x 2 per day) (inhibitor of mammalian target of rapamycin, I-mtor) and steroid; serum creatinin level was 20 mg/L as baseline. Patient was concomitantly treated with omeprazole 20 mg per day. CMV status was donor positive and receiver positive, but the patient did not received prophylaxis. Two months after transplantation, the patient presented a sepsis associated with diarrhea. The qPCR [RealStar CMV PCR Altona (CEIVD), Hamburg, Germany] was positive (6 log10) for CMV and he was treated for 6 weeks with valganciclovir, adapted to glomerular filtration rate (GFR) as recommended, allowing a prompt improvement (Fig. 1A). The viral load decreased to 4 log10 but was never undetectable. Viral resistance assessment was positive for the UL97 mutation (L595S). Viral load increased rapidly to 5 log10 and the patient was treated with foscarnet intravenously (50 μg/kg/12 h). After 5 days, the patient presented a foscarnet-induced acute kidney injury (AKI) requiring the discontinuation of treatment. The ANSM authorized the use of brincidofovir with renal adaptation doses (GFR = 20 mL/min/1.73 m^2^, creatinine = 38 mg/L). In accordance with CHIMERIX laboratory, an 80% decreased of the weekly dose was used. As recommended, the patient received 60 mg twice a week for 6 weeks starting August 20. In September 2015, creatinin level increased progressively to 58 mg/L (Fig. 1A) without any introduction of nephrotoxic drugs or a notable event in the medical history. Clinical evaluation was not relevant. Graft ultrasonography was normal, no overdosage of everolimus and tacrolimus was found, no proteinuria was observed, and graft biopsy was performed. Compared with surveillance biopsy performed after 3 months of transplantation that showed only vascular lesions (cv3 and ah1, Banff classification),^[5]^ the histological analysis revealed severe and extended acute tubular necrosis. Brincidofovir was discontinued and creatinin level decreased to 30 mg/L (Fig. 1A). In November 2015, CMV replication increased and CMV resistance assessment revealed a double population: a wild subset and a population associated with the UL97 resistance. The patient received intravenous ganciclovir allowing a partial decrease of the viral load and brincidofovir was reintroduced at 100 mg twice a week (adapted to renal GFR) during 6 weeks (Fig. 1A). Brincidofovir was efficient and viral load decreased from 5log10 to 2log10. Under brincidofovir treatment, the patient presented a novel episode of AKI. As suggested previously, graft ultrasonography and hydration statuses were normal. Medical history did not highlight any event or drug introduction. No overdosage of tacrolimus and everolimus was observed. Kidney graft biopsy was performed for the second time and found again severe extended acute tubular necrosis. Everolimus was discontinued and prednisone (20 mg) was introduced. Brincidofovir discontinuation allowed the renal function recovering to baseline (Fig. 1A). Brincidofovir-induced renal toxicity was retained as probable diagnosis (Fig. 1A and B). Application of the Naranjo probability scale revealed probable causality between brincidovir use and this AKI.^[6]^ Viral load was measured at 3.6 log10; CMV resistance assessment highlighted a mutation of UL54 (K513N) implying no use of cidofovir, brincidovir, and foscarnet. The patient was treated with artesunate (100 mg × 2 per day)^[7,8]^ during 14 days associated with Cytotect (anti-CMV gammaglobulin, 100 mL × 2 per day).^[9]^ This association failed to control CMV replication. Everolimus was stopped for leflunomide, 50 mg per day,^[10]^ and tacrolimus was tapered to 2 mg per day (0.02 mg/kg per day, T0 = 2 μg/L) despite the risk of allograft rejection. Three months later, CMV genome was not detected in blood samples. After 9 months of follow-up, CMV quantitative PCR in blood samples stay negative and creatinin level raised to 33 mg/L.

### 1.2 Case 2

The second patient is a 50-year-old man who received heart transplantation for valvular and ischemic terminal cardiomyopathy in January 2015. In addition, the patient presents a vascular chronic kidney disease with a creatinin level at 23 mg/L (CKD-EPI 40 mL/min/1.73 m^2^). He belongs to high-risk category with negative serological status for receiver and positive for donor (D+/R−) and received a 3-month prophylaxis using 450 mg valganciclovir daily. The initial immunosuppressive therapy included tacrolimus and steroid. He also received a prophylactic treatment using adapted doses of valganciclovir. In April 2015, the patient presented a CMV infection treated with intravenous ganciclovir followed by valganciclovir (Fig. 1C). In August 2015, patient was admitted for CMV replication without clinical sign of CMV disease, discovered on systematic follow-up (Fig. 1C). Although patient was treated, an increase of CMV viral load was observed by qPCR, suggesting for a potential resistance. UL97 mutation (H520Q) was identified and the patient was treated with Foscarnet intravenously to finally obtain undetectable levels of CMV DNA. Unfortunately, 5 days after discontinuation, the patient presented a foscarnet-induced AKI with an elevation of the creatinin level to 32 mg/L (Fig. 1C). No tacrolimus overdosage was observed. Renal ultrasonography was normal. After intravenous rehydratation, creatinin level returned to baseline. In September 2015, CMV viral load increased significantly (>3 log10), the human herpes virus 6 (HHV6) qPCR was positive, and we obtained a temporary authorization to use brincidofovir 100 mg twice weekly, adapted for his renal function, for the curative treatment of CMV infection. Concomitantly, everolimus was added for its properties against CMV. Brincidofovir treatment was efficient, but 2 weeks after the beginning, the patient presented an overdose of I-mtor and CNI complicated with AKI (creatinin: 52 mg/L, Fig. 1C). Blood samples showed a mechanic hemolysis with schizocytes and decreased haptoglobinemia associated with thrombopenia. The diagnosis of drug-mediated thrombopathic microangiopathy (TMA) due to conjugate overdose of everolimus and tacrolimus was retained. Both drugs were discontinued and a low dose of cyclosporine was introduced. In the absence of alternative treatment, CMV infection was treated with foscarnet, participating to the acute kidney injuring, requiring transitory intermittent hemodialysis. Foscarnet was stopped and creatinin level returned to baseline. During follow-up, a novel episode of CMV replication was observed in December, creatinin level was 23 mg/L (Fig. 1C). In the absence of other alternative, brincidofovir was reintroduced. Patient also received cyclosporin 50 mg twice a day (1.42 mg/kg per day) (C_0_ = 200 μg/L), and prednisone 5 mg. He was concomitantly treated with oral potassium for hypokaliemia, bisoprolol 10 mg per day, uradipil 30 mg twice a day. After 1 month, brincidofovir treatment allowed to inhibit CMV replication in blood. Unfortunately, the patient presented a novel episode of AKI (creatinin level raised to 44 mg/L, Fig. 1C) without overdosage of tacrolimus and everolimus. No particular event was reported in the medical history, and there was no proteinuria. Although hydratation status was normal, we observed a progressive increase of the creatinin level to 54 mg/L. This AKI was associated with persistent hypokaliemia associated with renal potassium leak, normoglycemic glycosuria, and tubular proteinuria (urinary ß2-microglobulin was increased). A kidney biopsy was performed (Fig. 1D). Here, we may show a severe proximal acute tubular necrosis associated with squeals of TMA lesions. After brincidofovir discontinuation, creatinin level decreased promptly to 33 mg/L (Fig. 1A). Application of the Naranjo probability scale revealed probable causality between brincidovir use and this AKI.^[6]^ In the following, patient presented 2 episodes of pneumonia with a decline of renal function. Seven months after, patient underwent extrarenal epuration. CMV genome is still undetectable in blood samples during the following.

## 2 Discussion

There are few alternative treatments for CMV-resistant infection. As previously described, prolonged exposition to ganciclovir, particularly with an insufficient dose, may promote UL97 mutation^[11]^ that lead to ganciclovir resistance. There is little alternative to treat UL97-mutated CMV infection. Cidofovir and maribavir^[1]^ are no more available in France and only foscarnet may be used, exposing patients to severe adverse events (genital mucosal damage, AKI). Letermovir is not yet available in France.^[12]^ Finally, promising results of cyclopropravir were recently published, but the molecule is still in development.^[13]^ The last options are potential combined therapies using artesunate,^[14]^ specifics anti-CMV immunoglobulin, requiring hospitalization. Use of leflunomide in addition to other immunosuppressive therapy may represent an additional treatment.^[10]^ Brincidofovir is also a possible alternative for curative and prophylactic treatment^[15]^ of emerging UL97 mutants. Moreover, its oral bioavailability allows an ambulatory treatment of complex infection. In comparison to cidofovir, known to promote proximal tubular nephropathy,^[16]^ this molecule exhibits a decreased renal toxicity. Safety and prospective studies did not show any renal adverse events. Here, we show that brincidofovir may induce severe acute tubular nephropathy in 2 specific patients. Indeed, our patients presented previously a chronic kidney failure and were exposed to other nephrotoxic drugs such as CNI. Causality of brincidofovir was considered as probable because, in both patients, we observed a relevant chronological history, a lack of other cause of tubular toxicity, a good outcome after drug discontinuation, and a positive reintroduction test in 1 case. In addition, indirect signs of tubular nephropathy such as glycosuria, aminoaciduria, and hypokaliemia with hyperkaliuria requiring oral supplementation were supporting this diagnosis. Although this severe side effect may lead to dialysis, brincidofovir-induced nephropathy seems to be reversible, even in chronic renal failure patients. As recommended by the manufacturer, brincidofovir requires a renal adaptation that was respected in both cases. These cases were reported to French national drug agency (ANSM), which considered this adverse drug reaction to be mentioned in brincidofovir monograph.

The precise mechanism of brincidofovir-induced AKI is not known and may need further investigations. Cidofovir-dihydrate was known to promote AKI^[17]^ due to its accumulation in renal tubule cells in an organic anion transporter 1 (OAT1)-dependent activity. Probenecid allowed reducing the risk by inhibiting OAT1 activity, avoiding accumulation. Brincidofovir is not a substrate of OAT1 but, by analogy to cidofovir, another anion transporter may be involved in the elimination of brincidofovir. Indeed, the coupling of cidofovir with a lipid tail allows especially to increase the intestinal absorption and to enhance the intracellular delivery.^[3]^ Moreover, safety studies were realized on healthy volunteers without a previous chronic kidney disease (volunteers must have a GFR >80 mL/min),^[3]^ known to induce tubular abnormalities, and without any concomitant nephrotoxic therapy such as CNI, known to interact with OAT.^[18]^

In conclusion, if brincidofovir is used to treated CMV-resistant UL97 mutants, clinicians have to be aware of drug-induced acute tubular nephropathy, especially in patients with chronic renal failure and/or concomitant use of nephrotoxic drug such as CNI.
