# Case report: Brincidofovir-induced reversible severe acute kidney injury in 2 solid-organ transplant for treatment of cytomegalovirus infection: Erratum

**DOI:** 10.1097/MD.0000000000006810

**Published:** 2017-04-28

In the article, “Case report: Brincidofovir-induced reversible severe acute kidney injury in 2 solid-organ transplant for treatment of cytomegalovirus infection”^[1]^, which appeared in Volume 95, Issue 44 of *Medicine,* the authors have requested a correction in Paragraph 1.1 line 23–24 where the recommendation should be changed from CHIMERIX to be in accordance with the French Society of Nephrology and Dialysis.

The authors state, “In the manuscript, we precise that doses adaptations were realized as recommended by CHIMERIX Laboratories. For Brincidofovir, renal dose adaptation, two guidelines are currently available. Guideline from CHIMERIX that only recommends to discontinue Brincidofovir when glomerular filtration rate is under 15 mL/min/1,73 m2. Guideline from the French Society of Nephrology and Dialysis (under the responsibility of ANSM) that recommends to discontinue Brincidofovir when glomerular filtration rate (GFR) is under 15 mL/min and, based on scientific publications, that suggests to decrease dose of brincidovir in the patient with chronic kidney failure.”

“The ANSM authorized the use of brincidofovir with renal adaptation doses (GFR = 20 mL/min/ 1.73 m2, creatinine = 38 mg/L). In accordance with CHIMERIX laboratory, an 80% decreased of the weekly dose was used. As recommended, the patient received 60 mg twice a week for 6 weeks starting August 20” has now been changed to appear as “CHIMERIX laboratory recommends to discontinue BCV under 15 mL/min/1.73 m^2^, but no renal dose adaptation was suggested in patients with chronic kidney failure. However, the French Society of Nephrology and Dialysis recommends the use of brincidofovir with renal dose adaptation. In accordance with both recommendations, the patient received 60 mg twice a week (GFR = 20 mL/min/ 1.73 m^2^, creatinine = 38 mg/L) for 6 weeks starting August 20.”

Figure 1 also appeared with two “D” labels when one should have been “B.”
